# A comparative analysis of dental education in China and Japan based on the four-component instructional design (4C/ID) model

**DOI:** 10.3389/fmed.2026.1843590

**Published:** 2026-07-20

**Authors:** Tianhan Pan, Jialu Cao, Weiwei Lin

**Affiliations:** 1 School of Stomatology, Zhejiang Chinese Medical University, Hangzhou, Zhejiang, China; 2 Faculty of Medicine, Dentistry and Pharmaceutical Sciences, Okayama University, Okayama, Japan; 3 The Stomatology Hospital of Zhejiang Chinese Medical University, Hangzhou, Zhejiang, China

**Keywords:** 4C/ID, cognitive load, curriculum comparison, dental education, undergraduate

## Abstract

**Background:**

Balancing cognitive load and clinical psychomotor skills within a limited curriculum time remains a core challenge in dental education. Faced with new healthcare demands driven by an aging population, dental education systems in both China and Japan require essential reforms. While current cross-national comparative studies mainly focus on macro-policy levels, introducing an objective educational model helps systematically analyze the core logical differences in micro-level pedagogical implementation and cognitive load management strategies. This study aims to examine differences in the distribution of 4C/ID components across Chinese and Japanese dental curricula, the localized strategies each country employs to refine cognitive load in dental education, and the feasibility of using the 4C/ID model as a retrospective curricular diagnostic tool to guide dental education reform.

**Objective:**

This study used a retrospective comparative case study design, analyzing comprehensive undergraduate syllabi and clinical practicum documents from Zhejiang Chinese Medical University (ZCMU, China) and Okayama University (OU, Japan). Using the Four-Component Instructional Design (4C/ID) model, all courses were mapped into four categories: authentic learning tasks (C1, clinical practice or simulation), supportive information (C2, subclassified into C2a: dental-specific theory and C2b: general/medical theory), procedural information (C3, instructor demonstrations), and part-task practice (C4, skill repetition). By combining semantic frequency analysis with the review of key pedagogical cases, this study assessed the instructional priorities of both countries.

**Results:**

Both curricula prioritize authentic learning tasks (C1) as a foundational means to foster professional competency, but differ in their structural approaches to modulating students’ cognitive load. Relying on a broad medical foundation, ZCMU front-loads general medical theory (C2b: 19.9%) while delaying dental-specific theory (C2a: 10.9%), concentrates clinical immersion in the last year (C1: 61.0%), and has limited skill training (C3+C4: 8.2%). In contrast, OU (C1: 39.7%) exhibits a longitudinal integration of C2a (15.9%) and C2b (24.3%) throughout the program, while dedicating considerably more hours to demonstrations and skill repetition (C3+C4: 20.1%).

**Conclusion:**

Analyzing the curriculum with the 4C/ID model offers valuable empirical insights that inform global dental education reform. To address the comorbidity challenges of a super-aged society, future dental reforms might incorporate lessons from Japanese and Chinese experiences by combining detailed theoretical scaffolding (C2) with comprehensive medical diagnostic reasoning to potentially manage cognitive load. Additionally, utilizing advanced digital simulation technology to initially separate procedural information (C3) and part-task practice (C4) from authentic learning tasks (C1) may offer a practical method to balance theoretical understanding with the development of complex psychomotor skills.

## 1 Introduction

In Health Professions Education (HPE), a key challenge is balancing the thorough acquisition of theoretical knowledge with the mastery of complex clinical psychomotor skills within limited curricula. Dental education exemplifies this challenge because it involves integrating substantial cognitive load with precise manual tasks within a confined anatomical space. As evidence-based medicine and the concept of integrated oral-systemic health have progressed, dental training has shifted from simple technical repetition to the systematic management of cognitive load within strict time constraints. Therefore, closing the gap between theoretical understanding and the development of psychomotor skills within a fixed instructional period has become essential for evaluating the effectiveness of dental programs (1). Ultimately, this effectiveness is confirmed externally through national licensing or board exams that verify graduates’ professional competence.

In East Asia, this challenge has driven two different, localized evolutionary paths. The stomatology model in mainland China, historically influenced by the former Soviet paradigm, anchors dental education within the larger framework of general clinical medicine (2). Under this integrated medical system, students undergo an extended period of general medical training before progressing to intensive, specialized dental coursework in the later stages of their degree program. In stark contrast, the Japanese model, heavily influenced by Euro-American paradigms, considers dentistry an entirely independent discipline, focusing on early specialization and rigorous, standardized pre-clinical assessments (3). This difference goes beyond just structural differences in degree programs; it fundamentally reflects distinct approaches to knowledge restructuring and professional skill development in the dental education systems of the two countries.

However, profound demographic transitions are currently compelling these established paradigms to undergo in-depth reform. As the first global pioneer to enter a super-aged society, Japan has accumulated substantial institutional assets in educating practitioners to manage geriatric dental care complicated by systemic comorbidities (4, 5). Concurrently, China is experiencing a rapid demographic transition toward an aging society. This shifting demographic structure and its associated healthcare demands place unprecedented requirements on the comprehensive clinical capabilities of dental practitioners. When this escalation in healthcare demands collides with traditional curricular architectures, dental students face significant challenges regarding competency attainment and allocation of cognitive resources. Consequently, systematically deconstructing and diagnosing existing dental education models, while drawing upon the successful experiences of mature systems, has assumed unprecedented value and urgency (6).

Although previous comparative studies have explored the macro-policy differences between the two countries’ dental education systems (7–9), the heterogeneity of their respective evaluation metrics has limited deeper investigations. Few studies have penetrated the foundational levels of dental school syllabi to reveal how these divergent educational philosophies translate into specific cognitive pathways during micro-level pedagogical execution.

To bridge this gap, it is essential to introduce a neutral and measurable analytical tool. Van Merriënboer’s Four-Component Instructional Design (4C/ID) model provides an ideal theoretical framework for this purpose. By deconstructing education into authentic learning tasks (C1, whole-task clinical experiences or simulation), supportive information (C2, cognitive scaffolding), procedural information (C3, step-by-step instructor demonstrations), and part-task practice (C4, repetitive psychomotor skill training), this model shows significant relevance in assessing the development of complex cognitive and clinical skills vital to dental education (10, 11). It also has the potential to quantify pedagogical strategies that were previously treated as a black box through reverse curricular diagnosis (12).

Building on this framework, this study addresses three interrelated research questions: (1) the specific differences in the micro-level configuration of these four instructional components between Chinese and Japanese dental curricula; (2) the underlying pedagogical logic regarding cognitive load management and clinical competency cultivation reflected in these structural disparities; and (3) the feasibility of using the 4C/ID framework as a retrospective diagnostic tool to refine curricular logic in response to evolving social healthcare demands.

This study selected the authors’ affiliated institutions—the School of Stomatology at Zhejiang Chinese Medical University (China) and the Dental School at Okayama University (Japan)—to quantify and compare their curricular architectures using the 4C/ID model. The objective is to quantitatively deconstruct the dental education models of both nations and, from the perspective of global medical education reform, to explore how optimizing curricular logic can mitigate the constraints imposed by external systemic pressures on the quality of professional training. The findings of this study provide an empirical basis for the glocalization of global dental education experiences and the restructuring of curricula to address the challenges posed by an aging population. Furthermore, it provides valuable insights into cognitive load management for similar disciplines within HPE that rely heavily on the mastery of fine psychomotor skills.

## 2 Materials and methods

### 2.1 Study design and data sources

This study employed a retrospective comparative case study design to quantify and contrast the undergraduate dental curriculum structures of two representative institutions: the School of Stomatology at Zhejiang Chinese Medical University (China; a 5-year stomatology program) and the Dental School at Okayama University (Japan; a 6-year dentistry program). These institutions were selected for their regional roles and academic significance within their countries. ZCMU is a key provincial medical university in China’s Yangtze River Delta and offers a 5-year stomatology program accredited by the Ministry of Education of PR China. OU is a leading national university and dental education hub in Japan’s Chugoku-Shikoku region, with a 6-year dentistry program aligned with the Ministry of Education, Culture, Sports, Science and Technology’s (MEXT) Model Core Curriculum for Dental Education. Both institutions focus on regional dental training and address societal issues such as an aging population. ZCMU has a Geriatric Oral Healthcare micro-specialty and integrates TCM to support oral-systemic health, while OU leads the Consortium for Dental Education Innovation in a Super-Aged Society (CODEE), pioneering aging-related curriculum reforms.

To ensure analytical objectivity and comprehensiveness, all raw data were extracted from official documents and publicly accessible pedagogical archives issued by the academic affairs offices of both universities. The collated textual corpus comprised the complete undergraduate syllabi and clinical clerkship curricula in effect at the start of the study (2025). These documents accurately reflect the established pedagogical frameworks and stable curricular structures that both institutions have consistently maintained in recent years.

### 2.2 Curriculum mapping and coding based on the 4C/ID model

To account for inherent differences between institutions in credit systems and individual session durations, all educational inputs across curriculum modules were uniformly converted to standardized 60-min instructional hours. These hours represent formal, scheduled faculty contact time and exclude estimated self-directed learning. This standardization was essential to mitigate institutional variability and included all lectures, laboratory practicals, preclinical simulations, and clinical rotations.

To contextualize the theoretical framework within the specific realities of undergraduate dental training, the foundational concepts of the 4C/ID model were first operationalized (Table 1). Based on these definitions, a specific coding taxonomy was established. Notably, to accurately capture the structural differences between dental professional instruction and broad medical foundations, Supportive Information (C2) was further subclassified into Dental-specific Supportive Information (C2a) and General/Medical Supportive Information (C2b).

**TABLE 1:** Conceptual definitions and operational mapping of the 4C/ID model in dental education.

4C/ID component	Original definition	Operational definition in dental education
Authentic learning tasks (C1)	Real-life, whole-task experiences integrating knowledge, skills, and attitudes for complex problem-solving.	Comprehensive clinical interactions with actual patients and high-fidelity, full-procedure simulations.
Supportive information (C2)	Cognitive scaffolding connecting prior knowledge to new, non-routine tasks (the “what” and “why”).	Theoretical frameworks act as cognitive scaffolds for diagnostic reasoning and clinical procedures.
Procedural information (C3)	Just-in-time, step-by-step instructions and rules for performing routine task aspects (the “how-to”).	Instructor-led demonstrations and standardized procedural guidelines for specific dental treatments.
Part-task practice (C4)	Highly repetitive practice of specific routine skills to achieve cognitive and psychomotor automaticity.	Self-directed, repetitive training to automate fine psychomotor skills.

**FIGURE 1:**
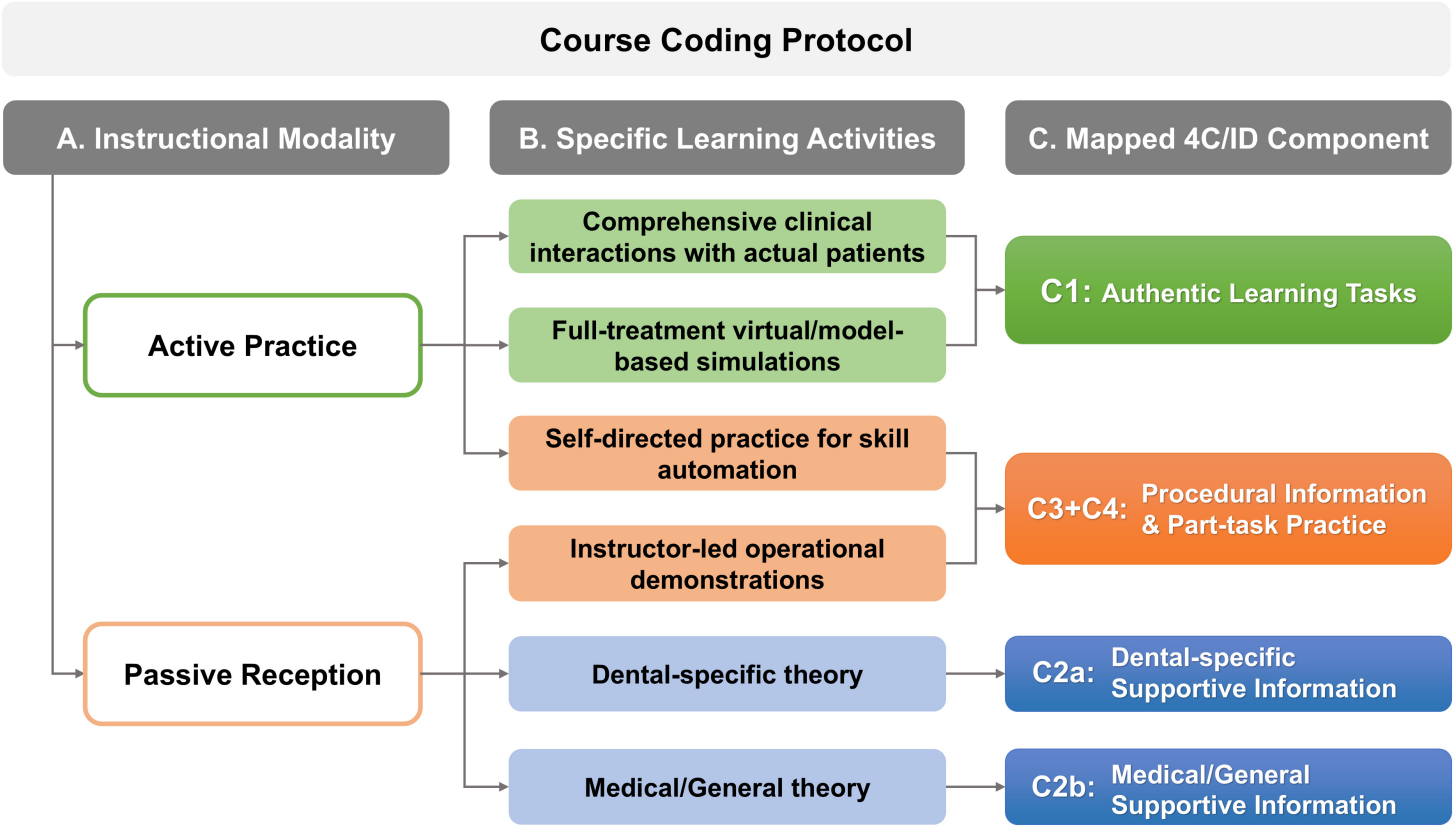
Flowchart of the curriculum mapping and coding protocol based on the 4C/ID model.

Following the standardization of instructional hours, two independent researchers retrospectively mapped all curriculum modules onto this established 4C/ID framework, guided by the pedagogical objectives detailed in the official syllabi (Figure 1). Coding discrepancies were resolved via consensus with a third senior expert, ensuring robust inter-rater reliability (Cohen’s Kappa > 0.80).

A preliminary review indicated that, constrained by the pragmatic realities of contemporary dental education, practical courses inevitably integrate instructor demonstrations (C3) with student-directed practice (C4) within single instructional units. Consequently, while acknowledging their distinct theoretical functions within the 4C/ID model, these two components were amalgamated into a single category (C3+C4) for the quantitative curriculum mapping. This methodological adaptation ensures an accurate reflection of the objective structural realities embedded within the current syllabi.

### 2.3 Semantic divergence analysis

To further elucidate the underlying pedagogical logic of the 4C/ID-based curricular designs, a semantic frequency analysis was conducted on the syllabi of both institutions. Initially, the relevant textual data were manually screened and collated. Subsequently, high-frequency core verbs and domain-specific nouns were extracted using R software (version 4.5.2). Specifically, Japanese texts were processed utilizing the *RMeCab* package, while Chinese texts were analyzed using the *jiebaR* package. To mitigate potential statistical biases arising from linguistic disparities and divergent tokenization algorithms, a custom-built Chinese-Japanese-English conceptual dictionary was constructed. Following the removal of stop words from all extracted tokens, semantically equivalent Chinese and Japanese terms—despite being segmented differently by their respective algorithms—were aggregated under a single, overarching English concept.

### 2.4 Data visualization

All data visualizations were performed using R software. The *dplyr* package was used to standardize the proportions of instructional hours across the 4C/ID categories and to construct a comprehensive term-frequency matrix for semantic analysis. To visually contrast the structural paradigms of the two institutions, the *ggplot2* package was employed. Additionally, to illustrate the longitudinal trends in curricular allocation across academic years, smoothed trajectories were generated using shape-preserving Monotone Hermite spline interpolation (the Fritsch-Carlson method).

### 2.5 Case analysis of representative pedagogical approaches

To complement the quantitative curriculum mapping, representative instructional modules were purposively sampled as qualitative case studies based on their structural significance and pedagogical distinctiveness. Guided by the 4C/ID theoretical framework, this textual analysis systematically evaluated detailed syllabus descriptions, teaching modalities, and instructional scaffolding strategies. By contextualizing the statistical data through these specific implementations, this qualitative phase aimed to comprehensively elucidate the underlying educational philosophies of both institutions.

## 3 Results

### 3.1 Authentic learning tasks (C1): differences in the proportion and distribution of instructional hours dominated by clinical clerkships

**FIGURE 2:**
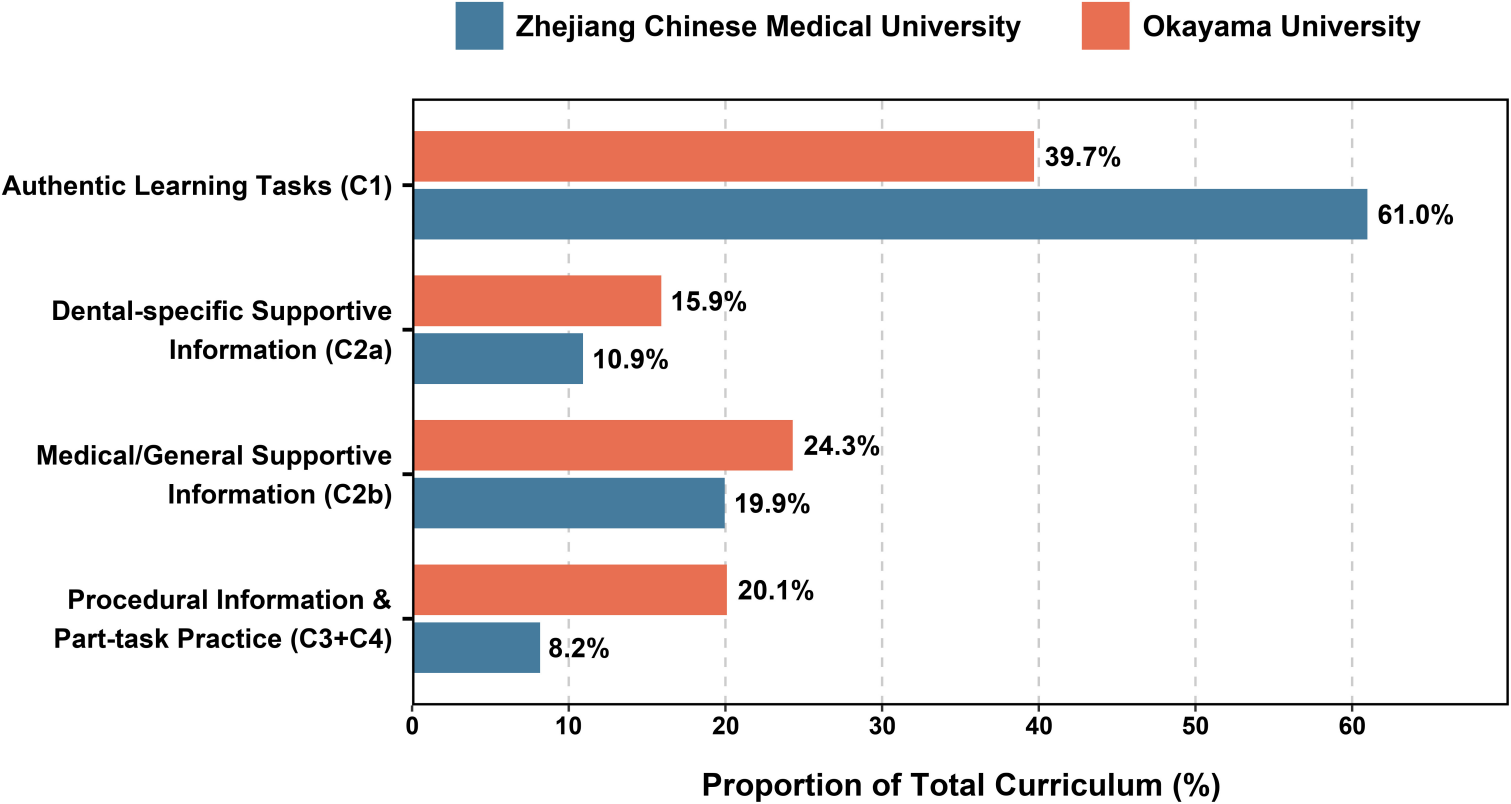
Comparative distribution of standardized instructional hours across 4C/ID components.

**FIGURE 3:**
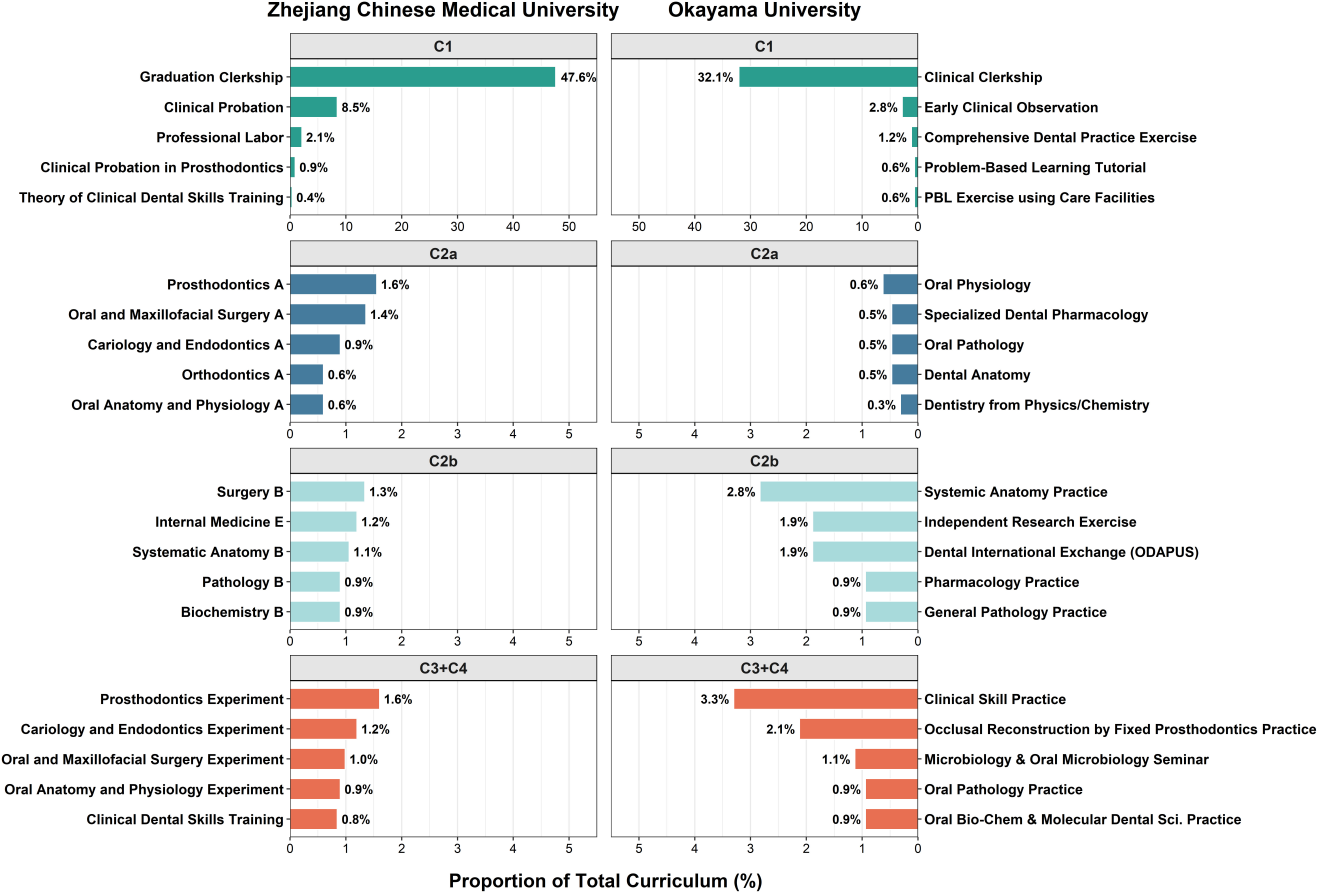
Granular decomposition of dominant courses within the 4C/ID model.

**FIGURE 4:**
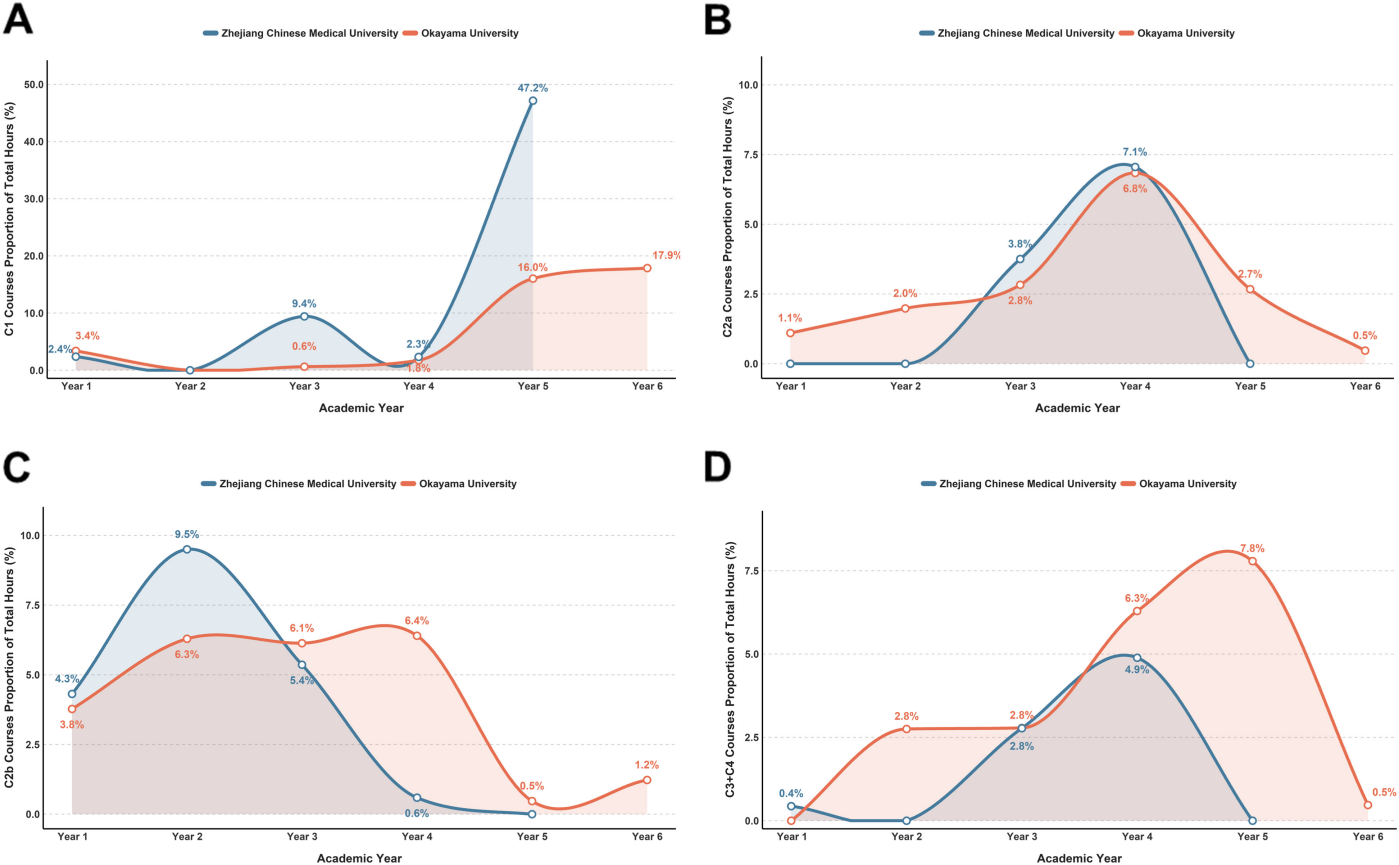
Temporal distribution of the 4C/ID curricular components across academic years. **(A)** Authentic learning tasks (C1). **(B)** Dental-specific supportive information (C2a). **(C)** General/medical supportive information (C2b). **(D)** Procedural information and part-task practice (C3+C4).

The two institutions show different structural approaches to allocating instructional hours for authentic learning tasks (C1) (Figure 2). C1 instruction accounts for 61.0% of total hours at ZCMU, but only 39.7% at OU. Despite this substantial disparity in proportions, both institutions mainly base their C1 on clinical clerkship courses (Figure 3). Besides the main clerkship component, ZCMU mainly assigns C1 hours to traditional, clearly defined clinical rotations in single disciplines, such as Clinical Probation in Prosthodontics. In contrast, OU tends to design C1 courses focused on clinical scenarios, with a strong emphasis on problem-based learning (PBL) integrated with interdisciplinary practice. On the timeline (Figure 4A), the C1 instruction at ZCMU follows a typical phased pattern, peaking in the third and final years. Meanwhile, OU’s C1 instruction also peaks in the final stages of the curriculum, but its extra year allows for a more even distribution of clinical clerkships across the last 2 years.

### 3.2 Supportive information (C2): phased theoretical transition and balanced longitudinal integration

Supportive information (C2) is further subdivided into specialized dental theory (C2a) and general medical foundations (C2b). The proportion of instructional hours allocated to these two modules at OU (15.9% and 24.3%, respectively) is substantially higher than that at ZCMU (10.9% and 19.9%) (Figure 2).

Zhejiang Chinese Medical University follows a clearly defined, phased pedagogical approach (Figures 4B, C). In the first two years, general medical foundations (C2b) take precedence, while specialized dental theory (C2a) is postponed until the third and fourth years, when it is delivered intensively. Starting in the fifth year during the clerkship phase, theoretical courses are discontinued, making room entirely for clinical clerkships. Specifically, ZCMU’s C2a courses are highly concentrated in a few core clinical specialties (Figure 3), including Prosthodontics (1.6%), Oral and Maxillofacial Surgery (1.4%), and Endodontics (0.9%). Meanwhile, ZCMU’s C2b courses are dominated by basic medical and clinical medical courses, such as Surgery (1.3%), Internal Medicine (1.2%), and Systematic Anatomy (1.1%).

In contrast, OU employs a balanced, longitudinal integration approach. As previously mentioned, the proportion of instructional hours invested by OU in both C2 modules exceeds that of ZCMU (Figure 2). Starting in the first academic year, OU simultaneously introduces medical foundations (C2b) and basic dental sciences (C2a)—such as Oral Physiology (0.6%), Dental Pharmacology (0.5%), and Oral Pathology (0.5%)—and integrates modules like scientific research and international exchange into C2b. This dual-track cognitive framework progresses smoothly throughout the entire 6-year program, maintaining relevant theoretical seminar courses even during the clinical clerkship phase.

### 3.3 Procedural and part-task practice (C3+C4): theory-based practice and independent skill training paths

Okayama University dedicates a substantial 20.1% of its instructional hours to skill training (C3+C4), greatly surpassing ZCMU’s 8.2% (Figure 2). Under the ZCMU framework, skill development is highly fragmented, usually existing only as concurrent, auxiliary laboratory sessions attached to theoretical instruction (C2a)—such as specialized modules like Prosthodontics Experiment and Cariology and Endodontics Experiment (Figure 3). In stark contrast, OU combines multiple individual practice exercises into standalone, comprehensive skill-based courses and includes highly contextualized, interdisciplinary training—such as Geriatric Prosthodontic Morphology Practice and Home Care Dental Practice—thus maintaining an independent and coherent pathway for skill development.

Chronologically (Figure 4D), because ZCMU’s part-task practice primarily serves as a subsidiary practical component of concurrent theoretical courses, the trajectory curve of its C3+C4 components closely aligns with that of C2a (dental-specific theory). By comparison, OU’s C3+C4 components maintain an independent, continuous skill-training trajectory throughout the degree program.

### 3.4 Semantic mapping and divergence of pedagogical priorities within the 4C/ID framework

**FIGURE 5:**
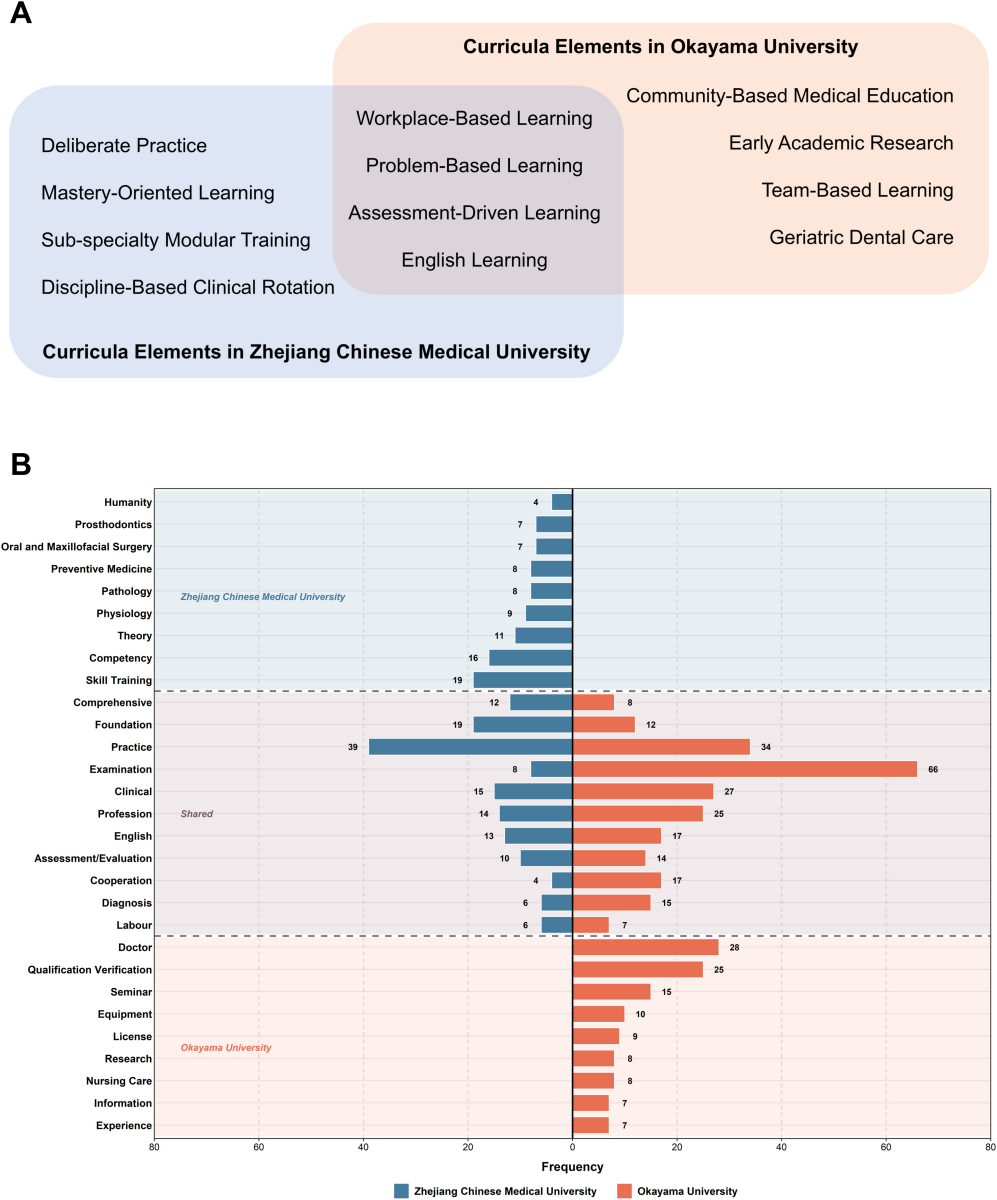
Qualitative mapping and semantic divergence of pedagogical priorities. **(A)** Qualitative mapping of shared and distinctive curricular elements. **(B)** Semantic frequency distribution of core terminology from official syllabi.

A comprehensive analysis integrating qualitative element mapping and semantic word frequency (Figure 5) revealed the shared principles and distinct priorities of the two institutions. Terms such as Practice and Clinical appear frequently in the syllabi of both institutions, reflecting a shared commitment to the core status of authentic learning tasks (C1). Simultaneously, both institutions integrate workplace-based learning (WBL) and English language instruction into their curricular architectures. Notably, Practice is the most frequently occurring word in the ZCMU syllabus (39 occurrences), whereas Examination appears most frequently in the OU syllabus (66 occurrences).

However, regarding the execution of theoretical knowledge and skill training (C2, C3+C4), the pedagogical divergence is highly pronounced. ZCMU emphasizes discipline-based clinical rotations and deliberate practice. Its high-frequency vocabulary is densely clustered around basic medical disciplines such as Physiology and Pathology; concurrently, the frequent occurrence of Oral and Maxillofacial Surgery and Prosthodontics aligns well with the high proportion of instructional hours dedicated to these two disciplines.

Conversely, OU’s high-frequency lexicon highlights terms such as Seminar, Research, and Cooperation, integrating community-based medical education, early academic research, and geriatric dental care into the C2 module. Furthermore, OU frequently mentions career and licensure-related terms such as Doctor, Qualification Verification, and License.

### 3.5 Case studies in 4C/ID model operationalization

Case studies further clarify the pedagogical practices of both institutions as they use the 4C/ID model. In practical skills teaching, ZCMU uses screen-based simulation (SBS) for pre-training. For example, before performing procedures such as inferior alveolar nerve block anesthesia and periodontal probing, students must watch simulated animations (Supplementary Figure 1). They must master standard operating workflows and identify anatomical landmarks within the SBS interface. Only after passing this simulation assessment can students perform live-patient operations. This digital simulation separates the step-by-step procedures (C3)—once taught by instructor demonstration and imitation—from part-task practice (C4). This separation may help manage the cognitive overload students face. It also avoids ethical constraints and shortages in clinical resources.

Regarding the scaffolding of Supportive Information (C2), the two institutions use distinct, localized strategies. OU’s curriculum has high granularity and contextualization. For example, while Pediatric Dentistry is one comprehensive course at ZCMU, OU divides it into modules such as Pediatric and Disabled Dentistry, Pediatric Coronal Restoration Practice, and so on. OU also offers interdisciplinary seminars as advanced cognitive scaffolds, encouraging students to connect theoretical knowledge with clinical tasks. Longitudinal research training throughout the program deepens students’ evidence-based reasoning. In contrast, ZCMU’s C2 scaffolding focuses on broadening dental knowledge. Its curriculum incorporates Traditional Chinese Medicine (TCM), introducing acupuncture and Tuina (massage) as adjuncts for oral mucosal and periodontal diseases, as well as mouth-breathing-related anomalies. Using digital platforms, ZCMU offers a Dental Clinic Management course that employs gamified simulations to support students’ understanding of entrepreneurship and clinic administration (Supplementary Figure 2).

## 4 Discussion

### 4.1 Divergent curricular architectures and their validation in national licensing examinations

This study used the 4C/ID model to compare the structural differences in undergraduate dental curricula between China and Japan. As shown by the quantitative mapping in this study (Figures 2, 4), the main difference is in the amount and organization of authentic clinical tasks (C1) and theoretical knowledge (C2). ZCMU allocates 61.0% of its credit hours to C1 courses and focuses on a concentrated, intensive clinical practicum before graduation. In contrast, at OU, C1 courses make up only 39.7%, with clinical tasks spread throughout the degree program, leaving a larger portion of curricular space for C2 courses.

This reflects the divergent strategies employed by the two institutions to manage students’ cognitive load and cultivate clinical reasoning (13). For OU, the gradual integration of theory and practice provides students with a continuous cognitive scaffold, helping to alleviate the psychological overload associated with early clinical exposure and enabling the steady development of clinical diagnostic and therapeutic reasoning. In contrast, ZCMU concentrates clinical tasks in a high-intensity clerkship during the final year; this model aims to expose students to a large volume of authentic cases in a short timeframe, supporting the progressive development of clinical psychomotor skills and mastery of clinical guidelines through repetitive practice.

Despite these structural differences, the effectiveness of both educational paradigms has been validated by their respective national competency assessment outcomes. Although the National Medical Licensing Examination (NMLE) in China and the National Dental Practitioner Examination in Japan differ vastly in candidate volume, both consistently maintain a pass rate of approximately 60%–70% (10). Available data show that both cohorts consistently exceed their national averages, confirming the adaptability of their distinct curricular designs. Specifically, ZCMU graduates have maintained a 100% pass rate on the NMLE practical skills component for multiple years, with a recent overall pass rate of 96.23% in 2024. Meanwhile, OU graduates have achieved similarly impressive results, maintaining pass rates between 92.2% and 95.7% on the Japanese national exams over the past 3 years (11, 12, 14). Graduates from both ZCMU and OU have achieved scores that significantly surpass their respective national averages, corroborating the adaptability of their curricular designs within their respective professional evaluation frameworks. Both models have optimized their targeted pedagogical outputs within the constraints of their localized institutional systems.

However, while licensure exam pass rates merely reflect the achievement of minimum competency standards, they are not synonymous with the cognitive load and acquisition difficulty experienced during instruction. The difficulty of skill acquisition in the Chinese model is primarily manifested in the structural competition between professional psychomotor training and an expansive foundational medical science curriculum. A multivariable analysis of the ZCMU curriculum shows that scores on the “Oral part” (practical skills) of the Chinese NMLE directly reflect the efficacy of and barriers to clinical skill acquisition. Empirical data confirm that improving the instructional quality of specialized stomatology courses and Stomatology X courses—which emphasize digital simulations—significantly improves Chinese dental students’ performance in practical assessments (15). This evidence not only helps delineate the thresholds for acquiring high-difficulty skills but also supports the utility of using digital simulation technology to decouple procedural information (C3) and part-task practice (C4) from complex, authentic whole-tasks (C1).

In the Japanese model, the pre-clinical Objective Structured Clinical Examination (OSCE) scores serve as an empirical proxy for the degree of skill acquisition. Research indicates that OSCE stations requiring high-level integration of technical maneuvers and communication impose a substantial cognitive load. Performance in these high-difficulty modules not only reflects bottlenecks in skill acquisition but also significantly predicts subsequent clinical clerkship quality and national licensing examination results (16, 17). Crucially, studies specifically targeting dental OSCEs have confirmed that despite high pass rates, 82% of dental students report elevated anxiety levels (18). This strongly reflects the potential cognitive burden inherent in acquiring complex clinical skills, highlighting a pedagogical orientation toward managing such cognitive load through refining instructional design prior to assessment.

### 4.2 Curricular differences in the context of historical and structural legacies

The divergence in curricular structures highlighted in this study mainly reflects the different design principles of dental education in the two countries, shaped by their respective national contexts. The development of the Japanese dental education system is largely driven by the mandatory 1-year postgraduate clinical training program introduced in 2006, which is fundamentally a systemic feedback mechanism that adapts to changes in healthcare needs and the demand-supply dynamics of the talent marketplace (19). This institutional change has led to a major shift in the goals of undergraduate teaching. Since mastery of clinical procedures is postponed until the postgraduate level, Japanese dental schools, such as OU, have been able to shift their teaching focus from immediate clinical automation to detailed knowledge building based on the 4C/ID framework. By breaking down the complex field of dentistry into specialized, scenario-based sub-courses, the Japanese educational model demonstrates a structured framework aimed at managing students’ intrinsic cognitive load, thereby potentially supporting the development of a solid and comprehensive theoretical foundation during their undergraduate years (1).

This institutional design of deferring clinical responsibilities creates ample space for early exposure to scientific research. By encouraging students to participate in research during their early undergraduate years, OU has sought to establish a genuine career-path experience system, helping students make more informed decisions about whether to pursue further academic studies or enter clinical practice (20). This structural alignment is indicative of a system that potentially alleviates the compensatory demand for postgraduate education, often exacerbated by information asymmetry—thereby implicitly aligning with a more stabilized expectation for advanced degrees among Japanese dental students (9). Furthermore, such a configuration may allow institutions to focus resources on high-fidelity simulation training and multi-faceted competency development, ultimately supporting preparation for the comprehensive demands of the Japanese national licensing exam (8, 21). This supports the findings of the previous word frequency analysis, where the frequent use of Seminar and Research in the OU syllabus underscores its emphasis on integration and inquiry, aligning closely with its larger share of C2 instructional hours.

**FIGURE 6:**
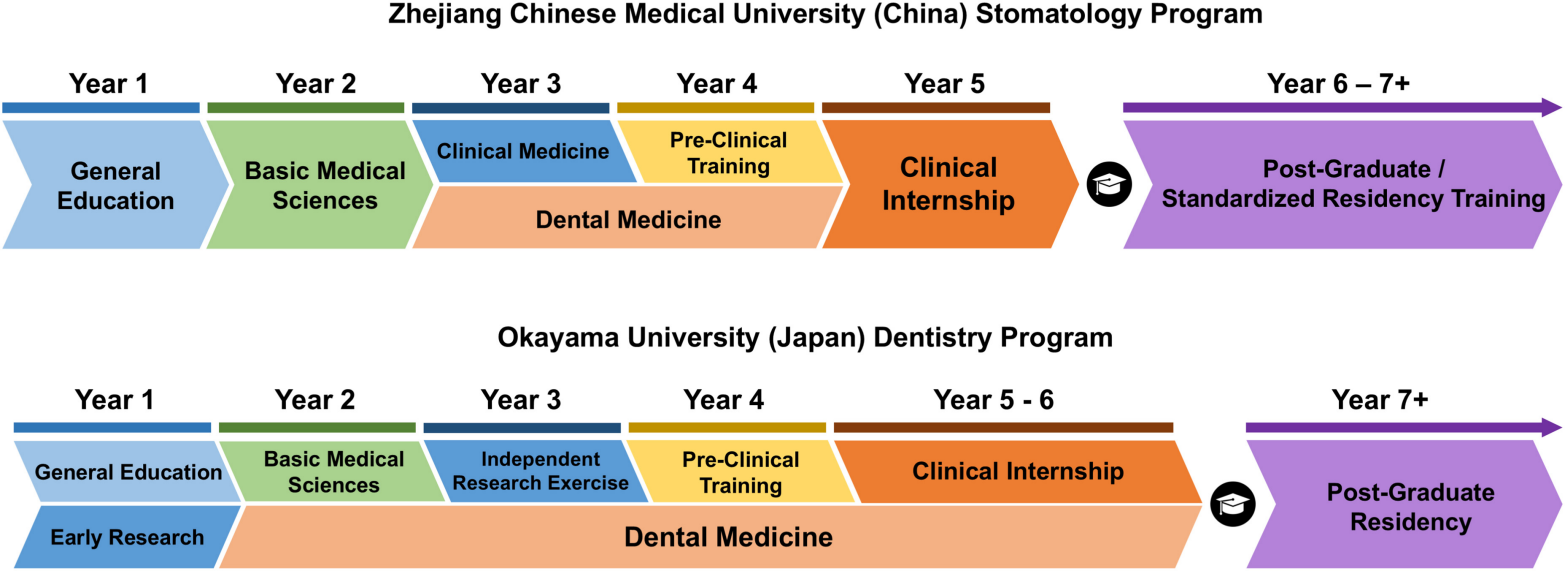
Macro-structural timelines of undergraduate dental education programs in China and Japan.

In contrast, ZCMU’s curriculum follows the Soviet medical education model, characterized by a three-stage linear progression: basic medicine, general clinical medicine, and stomatology (Figure 6). About 60%–70% of its initial coursework focuses on the systematic study of basic medicine (7). As our results indicate, ZCMU adopts a phased approach, heavily front-loading general/medical supportive information (C2b). Meanwhile, instructional hour allocations and semantic frequency analyses reveal that its dental-specific supportive information (C2a) is highly concentrated in Oral and Maxillofacial Surgery, Prosthodontics, and Endodontics. This architecture possesses strong local adaptability, aligning with China’s epidemiological realities and the primary healthcare system’s demand for generalist practitioners capable of independent practice upon graduation (22). Previous cross-national studies confirm that Chinese stomatology graduates possess broader skills for managing craniomaxillofacial surgical diseases and complex systemic conditions than dentists trained under the traditional North American model (23, 24). However, this overall structure entails pedagogical trade-offs: the significant early focus on general medical courses leaves fewer hours for specialized dental education and early clinical skills training (15). This also aligns with our quantitative findings that ZCMU allocates a smaller proportion of dental-specific supportive information (C2a) and skill-based training (C3+C4). Disciplinary silos within traditional curricula often lead to knowledge fragmentation. Coupled with current credential inflation and the intense pressure for further education faced by Chinese medical students (15, 25), postgraduate exam preparation and high-intensity clinical clerkships compete for limited cognitive resources during the final undergraduate year (26), exacerbating the competency shock students experience when transitioning to independent clinical practice (27–29).

To address current structural challenges, Chinese stomatology education is continually enhancing two areas: micro-level teaching methods and macro-level institutional systems. At the micro-pedagogical level, as mentioned earlier, ZCMU emphasizes a significant proportion of authentic learning tasks (C1) toward the end of the degree program and integrates digital virtual simulation technology into psychomotor skills training (C3+C4). This structural configuration represents a pedagogical approach oriented toward optimizing students’ practical skills and managing cognitive stress within the constraints of time and medical resources. At the macro-institutional level, China has systematically implemented the Standardized Residency Training (SRT) system over the past decade (9, 30). This training program, similar to Japan’s postgraduate clinical training, gradually shifts clinical exposure from the undergraduate to the postgraduate stage. Although there is still room for improvement, reforms such as SRT demonstrate a structural trajectory that may help balance complex psychomotor skills and cognitive load in Chinese stomatology education.

### 4.3 Practical implications for dental education reform

The comparative insights based on the 4C/ID model provide reciprocal implications for dental education reform in both countries. The contrast in the distribution of authentic learning tasks (C1) and the case analysis suggests that Chinese dental schools, while maintaining the volumetric advantage of high-intensity clinical training, could potentially benefit from further refining the methodology and quality of the transition from theoretical knowledge to clinical practice. Drawing upon the Japanese framework, fine-grained, contextually integrated supportive information (C2) courses can provide necessary cognitive scaffolds and structural transitions. For instance, broad stomatological disciplines such as oral and maxillofacial surgery or prosthodontics could be precisely decomposed into clinical-scenario-oriented sub-modules. Furthermore, addressing the structural dilemma Chinese medical students face between postgraduate entrance examinations and high-intensity clinical clerkships, introducing early research exposure and stratified career guidance mechanisms may provide a supportive framework that could tentatively foster the diversification of students’ long-term professional perspectives. This would moderately alleviate the crowding-out effect that highly homogenized competition for higher education exerts on cognitive resources during the clinical phase.

Conversely, the innovative experiences accumulated by Chinese institutions in addressing local challenges also offer highly valuable references for Japanese dental education. Methodologically, constrained by ethical reviews and medical resources, existing dental practices predominantly merge step-by-step demonstrations (C3) and repetitive practice (C4) into unified, instructor-supervised practical sessions. To address this status quo, the screen-based simulation (SBS) and virtual reality (VR) platforms adopted by ZCMU and other Chinese institutions offer plausible approaches for deconstructing C3 and C4. Given that ZCMU allocates only 8.2% of its curriculum to part-task practice, this digital pre-training may be viewed as a vital structural adaptation within this condensed curricular configuration. Implementing digital simulations could support students in safely developing both cognitive and psychomotor automation of procedural steps before their extensive clinical immersion. Based on this two-institution comparison, this approach offers a plausible reference for Japanese dental programs seeking to optimize simulation experiences with high psychological fidelity and part-task practice, all while avoiding high-risk clinical procedures. Furthermore, recent epidemiological evidence from Japanese hospital dentistry reveals that 86.5% of geriatric patients present with multimorbidity, underscoring the severe clinical complexity and the manifest risk of systemic complications in contemporary dental practice (31). Therefore, the Chinese model’s solid foundation in integrated internal medicine aligns well with modern oral-systemic health concepts. This systemic approach offers an informative pedagogical orientation that might assist in refining geriatric dental training in a super-aged society (4)—a need mutually corroborated by our qualitative mapping, which highlights “Community-Based Medical Education” and “Geriatric Dental Care” as distinctive C2 cognitive scaffolds within OU’s curriculum.

Notably, as non-native English speakers, medical students in both China and Japan rely heavily on English proficiency to support both further academic pursuits and scientific inquiry. The word frequency analysis in this study aptly reveals a shared focus on English instruction by both institutions. This finding objectively corrects the traditional perception that Japanese institutions relatively neglect medical English, indicating that medical schools in both countries acknowledge its importance in professional development (8). Recent quantitative research on ZCMU graduates also confirms that medical English is a critical foundation for enhancing dental graduates’ competitiveness in pursuing advanced degrees and conducting academic research (15). Therefore, in future curricular restructuring, it is urgent to introduce medical English training as a prerequisite cognitive scaffold as early as possible and integrate it throughout the degree program. This is anticipated to foster a supportive synergy with early research training, potentially enhancing students’ long-term professional competencies within a globalized healthcare context.

### 4.4 Methodological extension of the 4C/ID model as a retrospective curricular diagnostic tool and study limitations

This study extended the 4C/ID model to serve as a retrospective diagnostic tool for dental education, successfully quantifying abstract pedagogical priorities through semantic frequency analysis (32, 33). However, the trilingual translation involved in this cross-national application may inevitably introduce an interpretive bias.

Simultaneously, the divergent curricular architectures identified through the 4C/ID model urgently require further validation via multi-center, large-sample longitudinal cohort studies. By integrating multi-dimensional objective evaluation metrics—such as licensing examination pass rates, the caliber of admissions to advanced degree programs, and long-term career trajectories—future research can more precisely quantify and elucidate the sustained impact of pedagogical structural distributions on the quality of professional training. Furthermore, leveraging large language models (LLMs) tailored for the medical domain could tentatively assist in broader horizontal structural scans, offering a supportive framework to explore the potential longitudinal outcomes of various 4C/ID configurations on graduates’ clinical performance (34, 35).

## 5 Conclusion

This study used the 4C/ID model as a retrospective diagnostic tool, combining quantitative data with representative pedagogical cases to comprehensively compare the underlying architectures and cognitive load management strategies of undergraduate dental curricula in China and Japan. Both curricula aim to support a gradual transition from basic theory to authentic clinical environments, with the shared aspiration of helping graduates meet their respective national dental evaluation standards. Specifically, the Japanese model focuses on building progressive cognitive scaffolds (C2-dominant) to strictly control early cognitive load. In contrast, the Chinese model relies on a broad foundation in systemic medicine, emphasizing intensive, late-stage clinical immersion (C1-dominant). Integrating Japan’s scenario-based modular design with China’s systemic medical breadth and introducing digital simulations to safely decouple core skill training from complex clinical tasks draws on the advanced experiences of both models. This synthesis provides an empirical reference for optimizing global dental education systems, thereby better meeting the comprehensive demands for modern oral healthcare professionals in an aging society.

## Data Availability

The original contributions presented in this study are included in this article/Supplementary material, further inquiries can be directed to the corresponding author.
