# Supplementary material for: A comparative analysis of dental education in China and Japan based on the four-component instructional design (4C/ID) model
Source: Front Med (Lausanne). 2026 Jul 20;13:1843590. doi: 10.3389/fmed.2026.1843590 (PMC13430456; doi:10.3389/fmed.2026.1843590)
Supplement: Supplementary file 1 [file Table_1.docx]

Supplementary Material

# Supplementary Figures


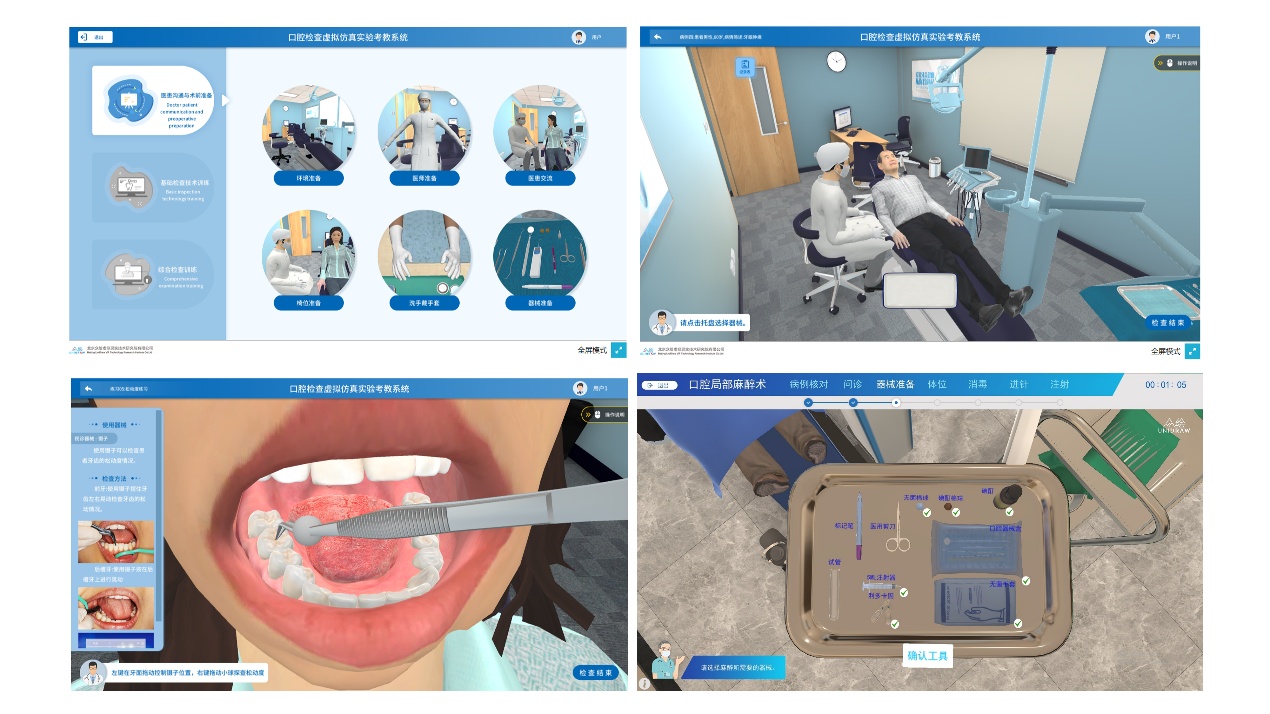


**Supplementary Figure 1.** Representative interfaces of the screen-based simulation (SBS) system for preclinical training of inferior alveolar nerve block anesthesia. The digital platform uses interactive 3D modules designed to help students master standardized local anesthesia workflows before live patient exposure. Information related to the course can be requested online at www.univrlab.com.


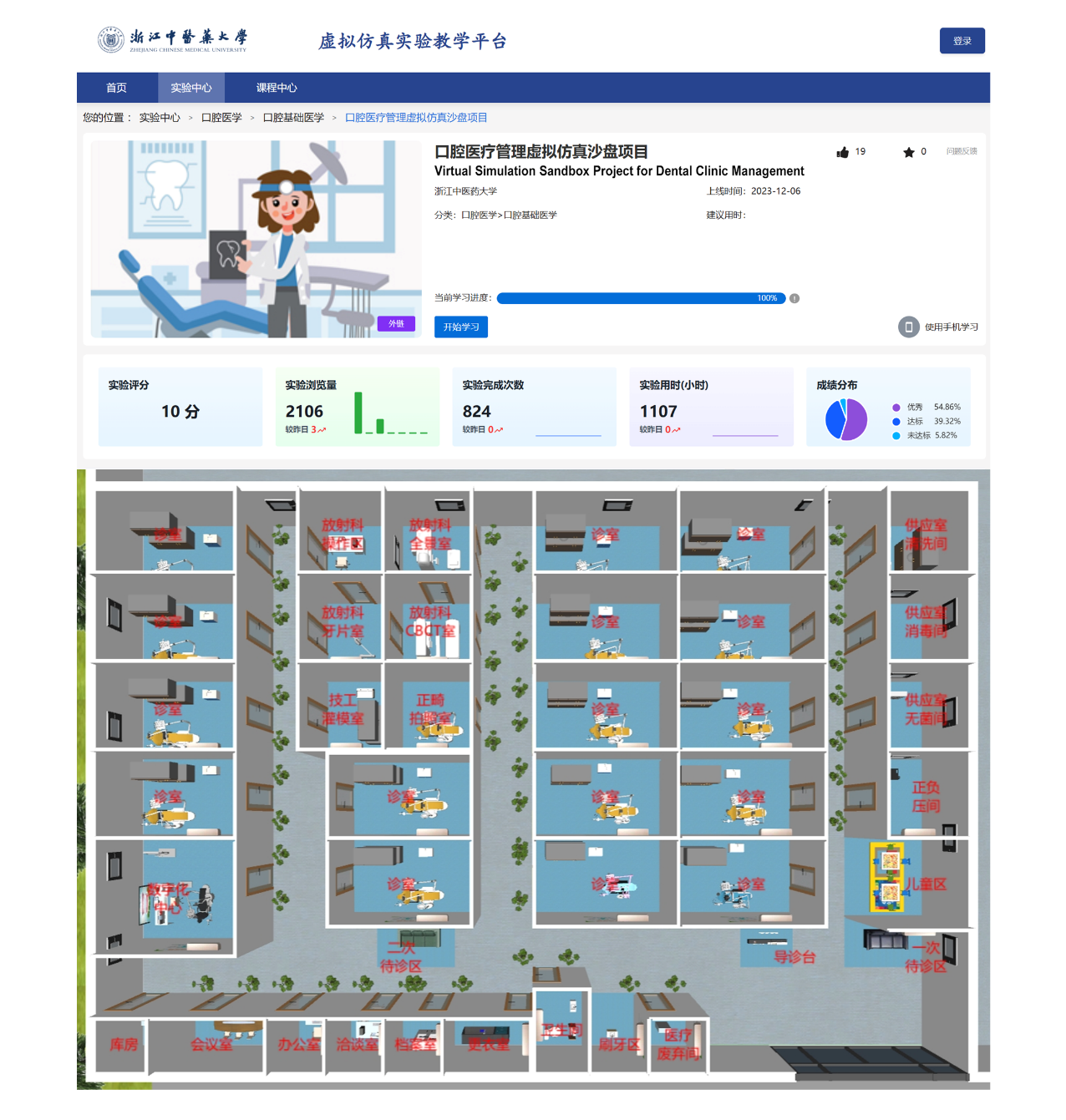


**Supplementary Figure 2.** Representative interface of the interactive Dental Clinic Management simulation platform. This top-down 3D architectural layout shows a detailed dental practice environment, allowing students to participate in spatial planning, functional zoning, and clinical administration. Information related to the course can be requested online at xnfzjx.zcmu.edu.cn/mengoo.
